# A smartphone-based diagnostic platform for rapid detection of Zika, chikungunya, and dengue viruses

**DOI:** 10.1038/srep44778

**Published:** 2017-03-20

**Authors:** Aashish Priye, Sara W. Bird, Yooli K. Light, Cameron S. Ball, Oscar A. Negrete, Robert J. Meagher

**Affiliations:** 1 Department of Biotechnology and Bioengineering, Sandia National Laboratories, Livermore, CA, 94550, USA; 2 Department of Systems Biology, Sandia National Laboratories, Livermore, CA, 94550, USA.

## Abstract

Current multiplexed diagnostics for Zika, dengue, and chikungunya viruses are situated outside the intersection of affordability, high performance, and suitability for use at the point-of-care in resource-limited settings. Consequently, insufficient diagnostic capabilities are a key limitation facing current Zika outbreak management strategies. Here we demonstrate highly sensitive and specific detection of Zika, chikungunya, and dengue viruses by coupling reverse-transcription loop-mediated isothermal amplification (RT-LAMP) with our recently developed quenching of unincorporated amplification signal reporters (QUASR) technique. We conduct reactions in a simple, inexpensive and portable “LAMP box” supplemented with a consumer class smartphone. The entire assembly can be powered by a 5 V USB source such as a USB power bank or solar panel. Our smartphone employs a novel algorithm utilizing chromaticity to analyze fluorescence signals, which improves the discrimination of positive/negative signals by 5-fold when compared to detection with traditional RGB intensity sensors or the naked eye. The ability to detect ZIKV directly from crude human sample matrices (blood, urine, and saliva) demonstrates our device’s utility for widespread clinical deployment. Together, these advances enable our system to host the key components necessary to expand the use of nucleic acid amplification-based detection assays towards point-of-care settings where they are needed most.

Arthropod-borne virus infections, including Zika, dengue, and chikungunya viruses, are on the rise globally. Dengue virus (DENV) and chikungunya virus (CHIKV) are already established in most tropical regions, while Zika virus (ZIKV) is rapidly spreading throughout Central and South America. ZIKV, like DENV, is an emerging mosquito-borne member of the *Flaviviridae* family. Since its discovery in 1947, ZIKV was believed to cause only mild illnesses, but the 2015 outbreak in Brazil revealed a link between the virus and severe fetal abnormalities, including microcephaly, congenital blindness, and fetal growth restriction, collectively referred to as congenital Zika syndrome^1^. More recently, ZIKV has been linked to the neurological disorder Guillain-Barré^2^, which can strike individuals at any age, leaving many population groups at greater risk if infected. While most arboviruses are thought to spread exclusively between the vector and the host, ZIKV has the capability to spread via bodily fluids, including blood, saliva, semen, and urine^3^; this may help the virus spread more rapidly than previously thought. Confounding this is the fact that most infected individuals remain asymptomatic, which presents additional challenges for limiting the spread of the virus. As the virus has made its way to the United States and has spread among human and mosquito populations, the need for rapid, low-cost diagnostics is even greater. Thus, there has been repeated emphasis by the World Health Organization (WHO) and U.S. Centers for Disease Control and Prevention to address the key limitations facing current infectious disease management strategies, especially in resource-limited settings^4^,^5^.

The current ZIKV outbreak has highlighted key limitations of existing diagnostic technologies. The fact that infections with Zika, dengue, and chikungunya viruses yield similar symptoms, including fever, rash, and joint pain, complicates differential diagnosis. Current serological and nucleic acid amplification tests are conducted on blood or other bodily fluids such as urine, saliva, and semen, and these tests require skilled technicians to extract, prepare, and load samples into complex analytical instruments^6^. Furthermore, systems capable of providing gold standard viral detection via nucleic acid amplification tests (NAAT)—typically multiplexed quantitative reverse-transcription polymerase chain reaction (qRT-PCR)—generally comprise bulky and complex peripheral components including expensive and power-hungry thermal cyclers (for rapidly heating and cooling samples) with built-in fluorimeter units (for performing real-time fluorescence detection). The associated turnaround time, complexity, form factor, power consumption, and cost of these devices make them sub-optimal for point-of-care applications, limiting their widespread deployment. Consequently, newly emerging NAAT devices are shifting from dedicated laboratory-based detection systems towards more compact, portable, and inexpensive field deployable systems^7^,^8^,^9^,^10^.

Reverse transcription-loop mediated isothermal amplification (RT-LAMP) has emerged as a popular isothermal NAAT for viral detection due to simplified thermal management and high sensitivity and specificity towards targeted sequences^11^,^12^,^13^,^14^. Isothermal NAATs eliminate the need for power hungry thermal cyclers, significantly lowering the complexity of hardware required for these tests. RT-LAMP detection of dengue^15^,^16^,^17^,^18^, chikungunya^19^, and other RNA viruses has been demonstrated previously, and at least one recent report has demonstrated RT-LAMP detection of Zika virus^13^. In most cases, these reports detect amplification with non-specific indicators of total DNA synthesis, such as monitoring turbidity^20^, observing the color-change indicator hydroxynaphtol blue (HNB)^17^, performing a post-reaction analysis involving addition of an indicator dye (*e.g*. SYBR Green)^21^, or performing gel electrophoresis to analyze the banding pattern^11^. Post-reaction analysis that requires opening the tube is undesirable, both for reasons of complexity and the risk of amplicon contamination, whereas non-specific indicators such as turbidity or HNB may be prone to detection of false-positive amplification events (a common, if under-reported problem with isothermal amplifications such as LAMP).

We address these issues by implementing RT-LAMP for Zika, dengue, and chikungunya in a closed-tube, multiplexable, target-specific format. The Zika and chikungunya assays utilize a recently developed detection technique that involves quenching of unincorporated amplification signal reporters (QUASR). Compared to most other reported LAMP detection modalities, QUASR offers very bright signals, greatly reduces the detection of false-positive amplifications, and offers the ability to multiplex two or more targets per reaction. These features reduce reagent costs and dilution when sample volume is limiting. QUASR is also fully compatible with complex sample matrices such as blood (up to 10%), which can interfere with some other detection modalities (*e.g*., SYTO intercalating dyes or pH-sensitive dyes). The use of QUASR in turn enables us to carry out the assays on a modular, wireless smartphone-operated platform powered by a 5 V USB charger. A custom smartphone application (app) controls a low-powered isothermal heating module and a multicolor LED excitation module via Bluetooth. The app also acquires images from the phone camera and processes them through a novel color and luminance-based detection algorithm capable of detecting multiplexed QUASR assay signals with greater accuracy than conventional image intensity analysis.

## Results

### Assay speed and sensitivity

We aimed to develop a rapid and reliable QUASR RT-LAMP assay for ZIKV, while relying on previously reported RT-LAMP assays for CHIKV and DENV^17^,^19^,^22^. We first set about identifying an optimal primer set for ZIKV, using the LAMP-compatible SYTO intercalating dyes to perform real-time monitoring. We then adapted the optimal primer set to the target-specific QUASR endpoint detection technique. The mechanism and chemistry of QUASR-based RT-LAMP detection techniques has previously been described in detail^22^. At least one recent publication describes a different RT-LAMP assay for ZIKV utilizing leuco crystal violet indicator^13^. However, the system can only detect total amplification and is incompatible with multiplexed assays. Furthermore, it is not clear how these assays perform with crude human bodily fluid sample matrices (blood and urine) or dry reagents, which are key features to improve the clinical relevance of a NAAT. Our QUASR RT-LAMP methods addresses these issues to enable robust and sample preparation-free ZIKV detection.

**Figure 1:**
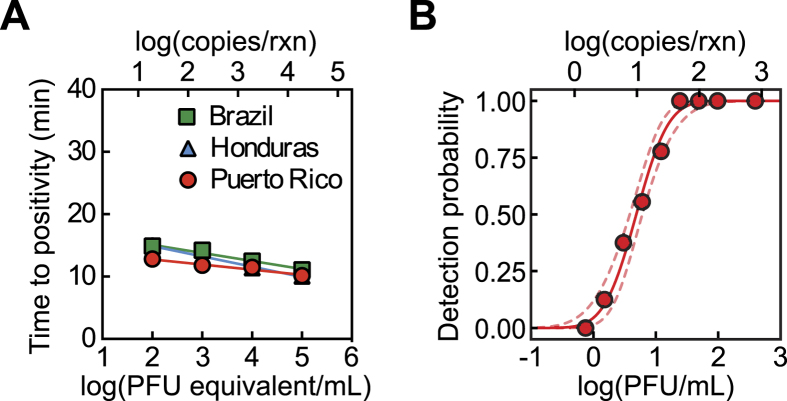
RT-LAMP rapidly detects Asian lineage strains of the Zika virus (ZIKV) with high sensitivity. (**A**) Positive amplification is detectable within 10 to 15 min using real time monitoring with SYTO 62, and reaction speed is comparable for RNA extracted from ZIKV isolates from Brazil, Honduras, and Puerto Rico (n = 6). (**B**) A plot of detection probability versus the concentration of intact ZIKV (Puerto Rico) in Tris buffer. Solid line represents curve fit by probit analysis, and dashed lines represent the 95% confidence interval. LOD_95  _= 2 PFU/mL, LOD_50  _= 4.9 PFU/mL (n = 12 to 36 replicates per dilution). Time to positivity is the Ct (cycle threshold) equivalent for LAMP reactions.

We prepared a ZIKV RNA standard from cultured ZIKV. We quantified ZIKV RNA genome copy number per plaque-forming unit (PFU) by qRT-PCR, using a synthesized DNA standard, and Vero cell plaque assays. Vero-cultured ZIKV was utilized for evaluating RT-LAMP assays throughout this study. We developed six candidate RT-LAMP primer sets that each targeted regions of the virus’ positive sense RNA genome that are conserved across the Asian lineage of ZIKV. Candidate primer sets targeted conserved regions within the Env, NS1, NS3, and NS5 genes and the 3′ untranslated region (3′-UTR). These regions were chosen empirically, based on the conservation observed in sequence alignments, although the NS5 (RNA-dependent RNA polymerase) and 3′-UTR are expected to be highly conserved. Furthermore, we have previously found viral polymerase and 3′-UTR to be good candidates for primer design^23^. We evaluated these six primer sets based on the speed with which they amplified their extracted ZIKV RNA targets and their tendency to engender false positives (Figure S1). The fastest amplifying primer set was also the most resistant to false positives (0/18 no template controls amplified) during an extended 75-minute isothermal incubation. This primer set, denoted NS5-8640, targets the ZIKV NS5 gene (the RNA polymerase). The NS5-8640 primer set performs optimally at 67 °C with 10 mM MgSO_4_ (optimization data not shown), detecting sample RNA concentrations of 10^5^-10^2^ PFU equivalent/mL (10^4^-10^1^ copies/rxn) in 10 to 15 minutes, respectively (Fig. 1). We then applied the QUASR detection method to RT-LAMP with the NS5-8640 primer set, labeling the BIP primer’s 5′ end with Cy5 and including a short (11 bp), complementary quenching probe with a 3′ Iowa Black RQ quencher. Upon cooling RT-LAMP reactions to room temperature, specific amplification of ZIKV resulted in bright fluorescence when excited by a red LED, while non-amplified reactions appeared completely dark. As expected, QUASR with Cy5 did not impact the speed or sensitivity of the NS5-8640 primer set for ZIKV, and QUASR was compatible with simultaneous real-time monitoring by SYTO 9, which fluoresces green upon intercalation into dsDNA. Previously described RT-LAMP assays for CHIKV and DENV were similarly rapid and sensitive in our hands compared to literature^17^,^19^. CHIKV RNA was detectable in 7 to 15 minutes at concentrations of 10^8^ to 10^3^ PFU/mL, and RNA templates from DENV 1–4 were detectable in less than 40 minutes (data not shown). In summary, QUASR in RT-LAMP with the NS5-8640 primer set rapidly and reliably detected ZIKV RNA.

A notable feature of LAMP and RT-LAMP is that it is possible to directly detect intact pathogens without first performing a nucleic acid extraction. In order to more fully characterize the sensitivity of the NS5-8640 RT-LAMP primer set for intact ZIKV, we ran a probit analysis on intact Vero cell-cultured virus serially diluted into Tris buffer. We found that the assay yielded a LOD_95_ (limit of detection) = 22 PFU/mL (44 copies/rxn) and a LOD_50_ = 4.9 PFU/mL (9.8 copies/rxn) (Fig. 1). We hypothesized that heating the viral sample before adding it into the RT-LAMP reaction mixture might lower the limit of detection by further disrupting viral structure and releasing RNA from the viral capsid. However, we found that heating ZIKV at 75 °C for 5 minutes in 10 mM Tris-HCl (pH 8.0) before adding virus to RT-LAMP had little impact on the LOD (Figure S3). We concluded that our RT-LAMP assay was suitable for direct isothermal amplification of intact ZIKV, which significantly simplifies the required sample preparation.

Rapid point-of-care tests are ideally shelf-stable for distribution in the absence of a cold chain. We prepared shelf-stable formulations of the QUASR RT-LAMP Zika assay by drying RT-LAMP reagents (except betaine, magnesium, and isothermal amplification buffer) along with a proprietary stabilization mixture from Biomatrica, Inc. These dry assays, rehydrated with a mixture of water, isothermal amplification buffer, and added magnesium, performed comparably to fresh reactions containing betaine (Figure S2). Real-time monitoring demonstrated positive detection of ZIKV as early as 10 minutes (actual reaction ran for 40 minutes), and endpoint discrimination by QUASR provided a positive to negative discrimination by fluorescence of 5–7 to 1 without background subtraction. In-house testing with several different QUASR RT-LAMP assays prepared with the Biomatrica stabilizers indicates that they remain stable at temperatures up to 40 °C for at least one month if protected from light (data not shown). The bright endpoint signal produced by QUASR permits detection by eye or simple optics, such as a smartphone camera. We therefore postulated that QUASR detection, coupled with assay stabilization in a dry format, would enable adaptation to shelf-stable assays that can be run at the point-of-care.

### Specificity of ZIKV primers

We evaluated the strain specificity and cross-reactivity of the NS5-8640 primer set against three ZIKV strains isolated from different regions of Latin America, genetically similar off-target flaviviruses, and off-target alphaviruses. QUASR RT-LAMP detected all three strains of ZIKV with identical speed and sensitivity (Fig. 1, Table 1). Asian lineage strains of ZIKV have no mismatches within the regions of the NS5 gene that the NS5-8640 primers target, so identical assay performance was expected. When QUASR RT-LAMP assays were tested against high levels of off-target RNA from other flaviviruses and alphaviruses, no amplification occurred (Table 1). Note that we did not test any African isolates of ZIKV with our assay, because based on sequence analysis of the 1947 MR766 isolate from Uganda, we identified >10 mismatches within the NS5 priming region and would not expect this isolate to amplify. For broad coverage of highly divergent RNA viruses, degenerate primers or multiple primer sets targeting different lineages is sometimes necessary for RT-LAMP or other NAATs. CHIKV and DENV primer sets were previously tested for cross-reactivity with DENV-1-4, Japanese encephalitis virus, WNV, SLEV, sindbis virus, or human RNA^17^,^19^. In this study, we also tested for cross-reactivity of these primer sets against extracted RNA from ZIKV (Puerto Rico), CHIKV, DENV-1, and WNV, and no amplification occurred.

### Detection of ZIKV in Human Sample Matrices without Sample Preparation

A sample preparation-free assay would dramatically simplify point-of-care diagnosis of ZIKV. RT-LAMP is able to operate for some viruses without an RNA extraction because the reaction temperature is high enough to allow primers and enzymes to access the viral RNA without performing a separate lysis step prior to adding the enzymes. The enzymes used in RT-LAMP are also resistant to inhibition by substances in clinical sample matrices. The no-extraction approach is fundamentally limited by the amount of sample that can be tested, and an extraction is still useful for increasing the concentration of viral RNA from a low-concentration sample, although at the expense of a more complex procedure.

**Figure 2:**
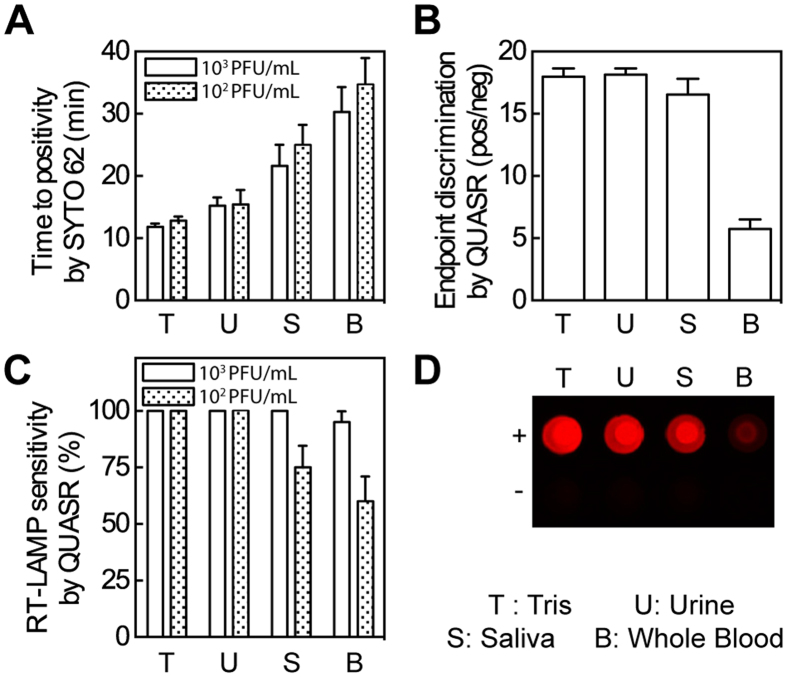
RT-LAMP robustly detects intact ZIKV in the presence of crude sample matrices. (**A**) Time to positivity (Ct equivalent for LAMP reactions), determined in real time by SYTO 62, is slowed in complex matrices. Error bars show standard deviation (n = 20). (**B**) QUASR detection enables clear and accurate endpoint discrimination in the presence of crude sample matrices, even whole blood, after cooling reactions to room temperature. Error bars indicate standard deviation (n = 31 to 40 per sample matrix, all positive results from A are pooled). While SYTO 62 signaled 1 false positive in urine no template controls (NTCs) and 7 false positives in saliva NTCs (n = 20 per sample matrix for NTCs), QUASR did not detect any false positives in NTC reactions. The criterion for a positive endpoint detection signal was determined from the NTC signals (Positive threshold value = Mean (NTC signals) + 3*Standard deviation (NTC signals)) which was calculated to be 1.06 based on the NTC values of saliva samples. (**C**) QUASR detection in RT-LAMP preserves sensitivity in crude matrices. Error bars indicate estimated standard error of proportion (n = 20). (**D**) Image of positive and negative ZIKV detection in sample matrices by QUASR RT-LAMP.

In the interest of developing the simplest possible assay protocol, we evaluated QUASR RT-LAMP’s capacity to detect ZIKV in crude human clinical matrices without lysis or extraction. We spiked intact Vero-cultured ZIKV into human urine, saliva, or blood to final concentrations of 10^2^ and 10^3^ PFU/ml, which are in the lower range of viral concentrations observed in specimens from Zika disease patients^24^. The ZIKV-spiked sample matrices were directly added at 10% (by volume) into the RT-LAMP reactions. Time to positivity, measured in real-time during ZIKV RT-LAMP reactions, slowed in the presence of crude matrices (Fig. 2A). However, the end point signal detected by QUASR clearly discriminated positives from negatives in urine, saliva, and blood (Fig. 2B). We detected ZIKV at or near a rate of 100% when it was present at a concentration of 10^3^ PFU/ml in clinical matrices. At a ZIKV concentration of 10^2^ PFU/ml, our detection rates in saliva and blood fell to 75% and 60%, respectively (n = 20) (Fig. 2C). Despite the reduced fluorescence intensity in the blood matrix compared to Tris buffer and other sample matrices, the difference between positive and negative samples by QUASR remained obvious (Fig. 2D). These results suggest that the QUASR RT-LAMP ZIKV assay is ideal for clinically relevant tests with minimum or no sample preparation.

We note that, although flavivirus RNA is routinely detected in the cell-free fraction of blood (plasma or serum) it is possible that whole blood would also contain intracellular viral RNA (e.g. from immature virions or virus replicating within white cells). Recent studies have given differing results on the distribution of dengue virus RNA within the fractions of whole blood (e.g. serum versus cellular components^25^,^26^). Our simple spiked samples may be imperfect models of clinical specimens from flavivirus infections. Curtis *et al*. have demonstrated extraction-free RT-LAMP amplification of HIV (an RNA virus that can be found in blood as free virions or intracellularly within T cells) from whole blood following a simple red blood cell lysis procedure^27^. Other procedures involving heat, buffer, or detergent-mediated blood lysis have also been demonstrated to be compatible with extraction-free LAMP detection of other intracellular and extracellular blood-borne pathogens^28^,^29^. Addition of such a procedure to our assay may improve recovery of intracellular viral RNAs (if present) with minimal added complexity to the protocol.

### A smartphone-enabled LAMP box

**Figure 3:**
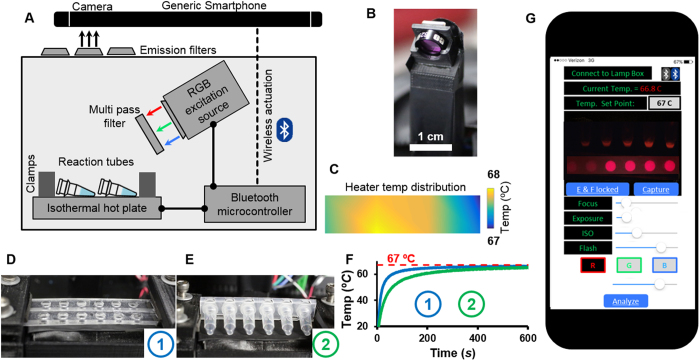
Smartphone enabled ZIKV detection. (**A**) Schematic of the RT-LAMP detection setup depicting the isothermal heater with reaction tubes, LED excitation source and Bluetooth microcontroller (Arduino Uno). (**B**) A 3 watt RGB LED coupled with an RGB multi band pass filter ensures a narrow excitation source for the assay reagents. (**C**) The isothermal heater provides a uniform surface temperature distribution within a 1 °C variation. The heaters can be loaded with either (**D**) off the shelf PCR polypropylene tubes or (**E**) custom made laser-cut reaction wells. (**F**) Thermal management and heat ramp rates are greatly improved with custom laser-cut wells. (**G**) The smartphone app wirelessly actuates the isothermal heater and RGB LED excitation source to enable real time monitoring and changing of the heater temperature along with illumination of the samples with appropriate excitation light source. The illuminated reagents are captured by the smartphone camera equipped with an interchangeable emission filter and the images are analyzed subsequently.

We developed a user-friendly, inexpensive and portable LAMP detection platform that leverages the robustness of our optimized ZIKV assay and the versatility of smartphones to enable in-field diagnostics (Fig. 3A). The complete NAAT device consists of three primary components: (i) a heating module, (ii) an assay reaction housing module, (iii) and an optical-detection/image-analysis module. Thermal management systems in commercially available thermal cyclers are often complex, owing to the installation of rapid ramp rate Peltier thermal blocks required for fast and precise thermal cycling. In contrast, isothermal NAAT devices exploit simpler heating setups. For example, we utilized a small isothermal heater powered by an ordinary 5 V power source. Our heater required very little energy (~85 mWh) to maintain a sufficiently uniform surface temperature profile for 40 min (Fig. 3C). Thermal cyclers for qRT-PCR applications work in conjunction with mass-produced and conventional reaction housing systems, typically thin walled polypropylene tubes (PCR tubes). Conversely, isothermal heaters often require custom reaction housings, such as microfluidic chips or modified PCR tubes, which can complicate sample loading or supply chain management^13^,^30^. We retained reaction housing simplicity by using off-the-shelf PCR tubes to carry out our LAMP reactions in our isothermal heater (Fig. 3D). Nevertheless, our heater is also adaptable to tailored reaction housing systems, such as custom laser-cut assay wells (Fig. 3E). We found that these shallow wells provided a larger thermal contact area of the fluid with the heated surface than PCR tubes did, enabling more rapid temperature equilibration within the reaction fluid (Fig. 3F). The optical detection modules in commercial NAAT are typically bulky and complex fluorimeters consisting of an excitation source, optical lenses, and a photon detector, typically a photo multiplier tube (PMT), a charge-coupled device (CCD) camera, or photo-diodes. These integrated detectors record the fluorescence signal emitted from the nucleic acid assay over time, and an inbuilt processor or a companion computer analyzes the fluorescence data. We have found that RT-LAMP-based amplification is more useful for yes/no end point determination than real-time monitoring at the point-of-care. First, real-time RT-LAMP tends to be quantitative over a narrower concentration range compared to qRT-PCR^23^. Furthermore, definitive binary end points (yes/no) are easier for non-experts to quickly interpret than quantitative real-time data. Our device contains a compact surface-mounted multicolored LED coupled with a multi-pass band filter to serve as an excitation source (Fig. 3B). Both the isothermal heater and the LED are actuated wirelessly (Bluetooth) via the smartphone application called “LAMPtoGo” (Fig. 3G).

Color changes in end point fluorescence detection techniques such as QUASR can be readily monitored by the human eye and a colored plastic filter. However, a digital photo sensor is more quantitative because properties like stereo-vision, visible color gamut, dynamic color range, and retinal photon sensitivity are parameters that vary from eye to eye^31^,^32^. Smartphones are ubiquitous and host a variety of sensors, including wireless technology (Wi-Fi, Bluetooth), global positioning system (GPS), and perhaps most significantly the CMOS (complementary metal-oxide semiconductor) optical sensor. The embedded camera unit coupled with advanced computing capabilities has made smartphones popular in bio sensing as an alternative to microscopes and photon detectors. The very familiar and intuitive application environment (Apple iOS and Android) cuts down the software learning curve and minimizes the need for new users to familiarize themselves with instrument operations. Several efforts have already harnessed the small footprint and versatility of smartphones to enable microscopy^33^,^34^, spectroscopy^35^, single molecule analysis^36^, colorimetry^37^,^38^,^39^,^40^, paper-based microfluidics^41^,^42^, and label-free detection^43^,^44^.

Our smartphone application takes advantage of the rapidly improving commercial CMOS sensor embedded in ordinary phones to acquire and analyze images of QUASR signals generated by our RT-LAMP assay more comprehensively than by the human eye. Inherent to all smartphones is the dynamic color balancing functionality, which is optimized for photography and thus introduces variability in the sensor parameters to adjust to the dynamic ambient conditions. This is not ideal for detection of assay signals, and various color calibration charts and reference assay samples have supplemented smartphone detection platforms to reduce such image-to-image variability. A 3D-printed “LAMP box” encloses our entire system assembly, blocking any ambient light and thus providing reproducible LED illumination. Moreover, the *LAMPtoGO* app overrides the auto-adjustment of camera parameters by the smartphone, granting manual control over the focal length, exposure time, and ISO of the CMOS sensor lens eliminating any variation in the emission signal. Once optimized for an assay, these parameters can be saved, allowing all subsequent assays to be performed with the same settings.

**Figure 4:**
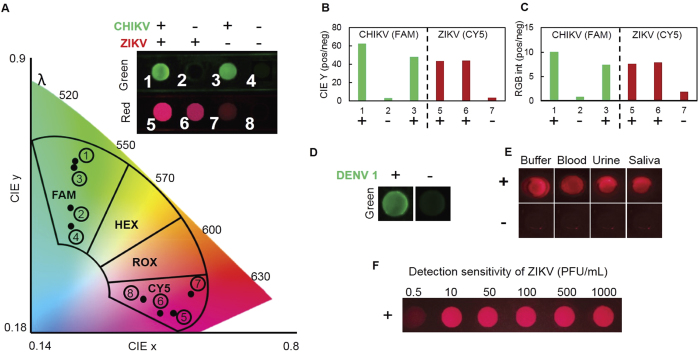
Mobile application for color sensitive multiplexed assay detection. (**A**) Duplex detection of ZIKV/CHIKV by QUASR RT-LAMP. 10 PFU/μL of each viral RNA was used in each reaction where indicated by a plus sign. No template controls are indicated with a negative sign. Samples were illuminated with Red/Blue light from the RGB LED and filtered images were acquired by LAMP2Go app. The analyzed images are then mapped over predefined fluorophore emission islands on the CIE xy chromaticity diagram, clearly distinguishing different viral target assays. (**B**) The positive to negative ratio of luminance value (CIE Y) for both FAM and CY5 assays are 3.2–6.4 times greater than that obtained from (**C**) RGB intensity analysis for the same sample (The ratio values were calculated for image # 1, 2, 3, 5, 6 and 7 with image # 4 and 8 taken as negatives for FAM and CY5 respectively). (**D**) DENV 1 was detected in a single assay containing SYTO 9 dye and combined primers for DENV1, DENV2, DENV3 and DENV4, with 10^3.4^ copies/μL of DENV1 viral RNA target shown here. The portable LAMP assay is able to function with (**E**) clinically relevant sample matrix with (**F**) similar sensitivity as a benchtop NAAT device. Intact ZIKV were used as targets in these assays.

We applied our smartphone-operated LAMP box to simultaneous detection of ZIKV, CHIKV, and DENV. We utilized the spectral multiplexing capability of QUASR to detect ZIKV and CHIKV in a single reaction, using primer sets labeled with Cy5 (far red) for ZIKV, and FAM (green) for CHIKV^22^ (Fig. 4A–C). In parallel, we detected DENV using a previously-reported assay for simultaneous detection of DENV-1–4^17^, adapted here for fluorescence detection with the intercalating dye SYTO 9 (Fig. 4D). We thereby demonstrated parallel detection of these three arboviruses with overlapping symptoms and epidemiology, and utilizing the smartphone platform for multiple detection modalities simultaneously (*i.e*. multiplexed QUASR, and non-specific intercalating dye). Since the CHIKV and DENV assays are based on previously published primer sets, we do not characterize them extensively as we did with our new ZIKV assay. We note that in multiplexing experiments with ZIKV and CHIKV, we were able to detect both targets when CHIKV was present in 10^5^-fold excess relative to ZIKV and vice versa, suggesting that QUASR multiplexing is capable of detecting coinfections. We also note that we have demonstrated serotype-specific detection of DENV-1 and DENV-2 using QUASR, spectrally multiplexed with ZIKV and CHIKV in two parallel reactions (not shown). It is not clear that distinguishing between serotypes of DENV is clinically useful in a point-of-care assay, versus a single assay for all DENV (such as shown here), as a patient with a positive test could be referred to a more sensitive and specific assay such as qRT-PCR for discrimination of serotypes.

For use within our smartphone system, we introduce a novel colorimetric detection algorithm, which analyzes the fluorescence images from the smartphone CMOS sensor. Such CMOS sensors consist of an array of pixel sensors coupled with a Bayer filter that passes red (R), green (G) and blue (B) light to select pixels. This type of RGB sensor is effective at reproducing a captured picture on the device display, but it is not ideal for colorimetric/fluorescence detection. Until now, endpoint fluorescence detection with assays such as QUASR has relied on the difference in the raw RGB intensities between positive and negative signals. However, intensity of fluorophores with emission wavelengths between red (700 nm), green (546 nm), and blue (435 nm) can be significantly underrepresented due to the coupling of the intensity and color data in the RGB color space, yielding significantly lower positive/negative signal values. Thus, for multiplexed fluorescence detection, it is useful to work in a color space that is capable of decoupling the color and the luminance of a pixel. This is achieved by transforming the acquired RGB pixel values to the International Commission on Illumination (1931 CIE) color space which resolves the image on the basis of two chromaticity color coordinates (CIE x-y values) and a luminance value (CIE Y value). The x-y chromaticity coordinates enable visual and unambiguous representation of multiplexed assays by mapping the QUASR signals onto predefined fluorophore islands corresponding to their emission wavelength spectra (Fig. 4A). Furthermore, the Y value (luminance), which is now independent of the color, increases the detection sensitivity of the CMOS sensor by 3.2–6.4 fold in comparison to the standard RGB intensity analysis (Fig. 4B,C). Our smartphone LAMP box performs comparably to benchtop thermal cyclers both in terms of sensitivity (detecting ZIKV at down to 100 PFU/mL) and the ability to detect ZIKV directly in a human bodily fluid matrix for clinical samples at realistic concentrations (Fig. 4E,F). Its small size and inexpensive design allows for widespread portable implementation outside the laboratory and at the point of need.

## Conclusion

We have developed a point-of-care NAAT device which combines our recently developed QUASR multiplexed RT-LAMP assay with a novel smartphone based detection system, allowing simultaneous analysis for ZIKV, CHIKV, and DENV which share overlapping symptoms and epidemiology. Our NS5-8640 primer set for RT-LAMP detection of ZIKV targets a highly conserved sequence within the Asian lineage of ZIKV, yielding high specificity with no cross reactivity with other genetically similar flaviviruses and alphaviruses. Our recently developed QUASR RT-LAMP detection chemistry coupled with the smartphone image analysis application enables unambiguous detection, yielding performance similar to benchtop NAAT devices. The ability of our LAMP reagents to function in dried formats eliminates cold chain requirements during shipping/storage. Our system can work directly with human samples (blood, saliva, urine), which minimizes the necessity for complex sample prep and simultaneously incorporates the versatility of an ordinary smartphone to provide an alternative to PCR to advance molecular diagnostics at the point of care.

## Methods

### Cells and Viruses

We found that ZIKV cultured from Vero cells had a titer of 1.6 × 10^7 ^PFU/mL and an RNA copy number of 2,000 copies/PFU. ZIKV cultured in insect (C6/36) cells had a titer (evaluated in Vero cells) of 2.2 × 10^8^ PFU/mL and an RNA copy number of 90 copies/PFU. Vero cells were maintained in Minimum Essential Medium alpha supplemented with 10% (vol/vol) fetal bovine serum, 100 μg/ml penicillin, and 100 units/ml streptomycin (Life Technologies) at 37 °C under 5% CO_2_.

ZIKV strains PRVABC59 (Puerto Rico) and R103451 (Honduras) were obtained from Brandy Russell (Centers for Disease Control, Fort Collins, CO). The Brazilian strain was provided by Michael Busch (Blood Systems Research Institute, San Francisco, CA). All strains were propagated in Vero cells. Briefly, cells were infected with virus at a multiplicity of infection (MOI) of 0.01 and supernatant was collected after 7 days. Particle forming units (PFUs) were determined using a standard plaque assay consisting of an agarose overlay and crystal violet staining on Vero cells.

### ZIKV target preparation

ZIKV RNA was isolated using QIAamp Viral RNA Mini extraction kit (QIAGEN) following manufacturer’s protocol. 140 μl of viral supernatant from strain PRVABC59 was used for the extraction along with yeast tRNA (Thermo Fisher) as a carrier.

The ZIKV (PRVABC59) was spiked and further diluted in 10 mM Tris-HCl (pH 8.0) or human matrices (whole blood, saliva or urine) to various virus concentrations. Human sample matrices pooled from de-identified healthy donors were purchased from a commercial supplier (Innovative Research, Novi, MI). The matrices were tested by the supplier using FDA-approved methods and found negative for blood borne pathogens including HIV, HBV, HCV and syphilis. The supplier provides no identifying information about the donors, making our use of the sample matrices exempt from human subjects research regulations under US 45 CFR 46.101(b). Thus our study does not require a formal institutional review board approval.

### RT-LAMP primer design

Candidate regions for primer design were identified by performing an alignment with complete genomes of 17 Zika virus isolates from the Asian/Latin American outbreak lineage. Alignment was performed with Clustal X2 software using default parameters, and scanned visually for regions of high conservation. LAMP Designer software (Premier Biosoft) was used to identify candidate primer sets for regions of high conservation (typically using an input range of 300–500 bases identified from software), using the software’s default parameters for primer design. For each target region, the software produces a list of candidate sequences ranked by quality score. For each group of candidate primers, the primers were manually subjected for further tests (Supplementary Information).

### RT-LAMP assay

RT-LAMP reactions were carried out in PCR tubes or 96-well plates with 1 μL of the sample in 9 μL of RT-LAMP reaction solution. The fresh reaction mixture contained 1x Isothermal Amplification Buffer (20 mM Tris-HCl, 10 mM (NH_4_)2SO_4_, 50 mM KCl, 2 mM MgSO_4_, 0.1% Tween^®^ 20, pH 8.8@25 °C, NEB), 700 mM of Betaine (Sigma), 0.14 mM of each nucleotide, 8 mM MgSO_4_, 2 μM SYTO^®^ 9 Stain (Thermo Fisher Scientific), 2 units of Porcine RNase Inhibitor^45^, 0.2 μM each F3 and B3, 0.8 μM each LF and LB, 1.6 μM each FIP and BIP primers (Table S1), 3.2 units of avian myeloblastosis virus (AMV) reverse transcriptase (Life Sciences Advanced Technologies Inc., St Petersburg, FL), and 3.2 units of Bst 2.0 WarmStart^®^ DNA Polymerase (NEB). To make the dried reagent formulation, isothermal amplification buffer, betaine and magnesium sulfate were removed. While rehydrating, isothermal amplification buffer and magnesium sulfate were added to reagents dried along with a proprietary stabilization mixture from Biomatrica Inc (Supplementary Information).

For real-time monitoring of the reaction, fluorescent intensity was collected every 1 minute through FAM channel during incubation at 67 °C for 40 minutes and for the end-point, fluorescent intensity was measured at room temperature though Cy5 channel after the incubation using CFX-96 real-time thermocycler (Bio-Rad Laboratories, Hercules, CA). For the RT-LAMP on the box, each laser cut custom well was loaded with mixture of 2.5 μL of the sample and 22.5 μL of the RT-LAMP reaction solution and then placed on the isothermal heating block at 67 °C for 40 minutes and imaged at room temperature as described below. The volume of the laser cut wells were designed to be 25 μL to allow for a larger top cross-sectional imaging area (Dia. ~4.6 mm). The larger cross-sectional also served to increase the heat transfer between the reaction fluid and the isothermal heater.

### Specificity and sensitivity of RT-LAMP

For testing specificity of the RT-LAMP primers, each Zika stain was serially diluted to 100, 10, 1, and 0.1 PFU/mL in 10 mM Tris-HCl (pH 8.0) and used as a template for the RT-LAMP reaction described above. For testing cross-reactivity with other members of the flavivirus group and alphaviruses group, at least 100 PFU equivalent purified RNA from each virus was used as a template.

The sensitivity of the RT-LAMP assay for the detection of ZIKV was determined by Probit analysis. Briefly, the virus (PRVABC59) was tested with 2-fold serial dilution in replicates at each virus concentration. The detection probability with 95% confidence interval was obtained by statistical analysis using probit regression model with R (version 3.3.1).

### Portable LAMP box

The isothermal heater and RGB LED (3 W RGB tricolor; Digi-key; Part # 516-2246-1ND) fitted with a triple-bandpass filter (Edmund Optics #87-237) were operated via an Arduino UNO R3 microcontroller (ATmega328, assembled) which communicated to the smartphone wirelessly through a HM11 Bluetooth low energy (BLE) module (Seeed Technology Co., Ltd). Two 10 W, 3.3 Ω ceramic wire wound resistors (Digi-key; Part # 3.3W-10-ND) in series functioned as heaters by converting electric current to heat. One of the Arduino’s digital pin provided the heater with 5 V input voltage through a N channel MOSFET (MOSFET N-CH 60 V 55 A TO-220; Digi-key; Part # 497-6742-5-ND). A temperature sensor (Digi-key; Part # AD22100STZ-ND) monitored the heater temperature, and the signal was sent to one of the analog pins in the microcontroller. This analog reading was converted into temperature which was continuously monitored to operate the heater isothermally at the desired set point via a pulse width modulation (PWM) signal. The PWM pin outputs 100% of its signal until the temperature of the heater reaches the set point and shuts down the current to the heater if the temperature exceeds the set point. The Arduino program was written and compiled in Arduino 1.6.6 integrated development environment (IDE). A separate function library was created to enable communication between the smartphone and the Arduino via the BLE module. A dark plastic enclosure for the setup (LAMP box) was designed using FreeCAD and 3D printed using MakerBot Replicator 2X.

The embedded Bluetooth module in the LAMP box can create a seamless communication interface between the device peripheral components (isothermal heater and RGB LED source) and any smartphone via our custom developed application called *LAMPtoGo*. The app is quite intuitive with a simplistic GUI consisting of three main screens, namely (i) control panel screen, (ii) sample selection screen and (iii) Colorimetric-Luminance image analysis screen (SFIG 4). Once the app is launched, the control panel screen allows the user to establish a Bluetooth connection with the LAMP box, set the heater temperature and monitor the real time heater temperature profile. It also displays a live camera feed and allows the user to override the default smartphone camera parameters. This enables the users to manually tune the focal length, exposure and ISO of the camera lens independently. The ability to digitally zoom images via the apps affine transformation framework enables a total of 6 X magnification without noticeable pixel degradation. Images are acquired through an array of fluorescence filters positioned in the box (Edmund Optics #87-777 and #62-979 for FAM and Cy5, respectively). The user can also actuate inbuilt smartphone flash LED at varying intensities to increase/decrease visibility within the box. After running the LAMP reaction and image acquisition, the user is taken to “sample selection” screen where they can select multiple regions of interest within the acquired images using touch gestures for post processing. After doing so, the results are displayed on the final “Colorimetric-Luminance image analysis” screen.

### Colorimetry-luminance based image analysis

The raw image data from the CMOS sensor is stored as a bitmat data file containing 4 bits per pixel (one for each red, blue, green and alpha pixel values) corresponding to standard RGB (sRGB) color space. The alpha channel which measures pixel transparency is discarded as it is constant for all pixels. A series of transformations is applied pixel matrix to convert the sRGB values to the corresponding CIE xyY color space as depicted in SFIG 5. Gamma transformation is applied with a gamma value of 2.4 to account for the non-linear intensity scale of the CMOS sensor^46^. The averaged chromaticity values (x, y) are then mapped on a 2D chromaticity diagram with predefined islands corresponding to the emission spectra of four spectrally different fluorophores (FAM, HEX, ROX and CY5). The boundaries of these island are determined by the emission wavelength of the fluorophore and the color gamut of the device. The third parameter, luminance (Y) contains information about the strength of signal irrespective of the color. Finally, the results can be viewed within the app as plots or exported as.csv files for further analysis. The time stamp and GPS location is also exported for each run for archival.

## Additional Information

**How to cite this article:** Priye, A. *et al*. A smartphone-based diagnostic platform for rapid detection of Zika, chikungunya, and dengue viruses. *Sci. Rep.* **7**, 44778; doi: 10.1038/srep44778 (2017).

**Publisher's note:** Springer Nature remains neutral with regard to jurisdictional claims in published maps and institutional affiliations.

## Supplementary Material

Supporting Information

## Figures and Tables

**Table 1:** Viral RNA used for testing sensitivity and specificity of NS5-8640 RT-LAMP primers.

Virus	Strain or isolate designation	Primer reactivity	Target concentration (PFU equivalent/mL)
*Flaviviruses*			
Zika virus (ZIKV)	PRVABC59 (Puerto Rico)	+	10^2^–10^5 (1)^
	Brazil	+	10^2^–10^5^
	R103451 (Honduras)	+	10^2^–10^5^
St. Louis encephalitis virus (SLEV)	KERN217	−	10^5^
West Nile virus (WNV)	L-CA-04 SAC-04-7168	−	10^7^
Yellow fever virus (YFV)	17D	−	10^7.1^
Dengue virus serotype 1 (DENV-1)	BC-796	−	10^6.4^
Dengue virus serotype 2 (DENV-2)	BC-122-94	−	10^5.9^
	Jamaica/N.1409 accession number M20558.1	−	Ct 24 min^(2)^
Dengue virus serotype 3 (DENV-3)	BC 156-97	−	10^5.5^
*Alphaviruses*			
Chikungunya virus (CHIKV)	Ross	−	10^8.5^
Western equine encephalitis virus (WEEV)	KERN 5547	−	10^5^
Venezuelan equine encephalitis virus (VEEV)	TC-83	−	10^9.5^

^1^We tested a larger range of ZIKV target concentrations in other experiments presented in this paper.

^2^DENV-2 isolate JMP1402 was not quantified by plaque assay. Extracted RNA was quantitated by RT-qPCR and used undiluted as a template in RT-LAMP. RT-qPCR Ct value is indicated.
