# Supplementary material for: A smartphone-based diagnostic platform for rapid detection of Zika, chikungunya, and dengue viruses
Source: Sci Rep. 2017 Mar 20;7:44778. doi: 10.1038/srep44778 (PMC5357913; doi:10.1038/srep44778)
Supplement: Supporting Information [file srep44778-s1.pdf]

Supporting Information (SI)

A smartphone-based diagnostic platform for rapid detection of Zika, chikungunya, and dengue viruses

Aashish Priye[a], Sara W. Bird[a], Yooli K. Light[b], Cameron S. Ball[a], Oscar A. Negrete[a] and Robert J. Meagher[a]

[a]Department of Biotechnology and Bioengineering, Sandia National Laboratories, Livermore, CA 94550

[b]Department of Systems Biology, Sandia National Laboratories, Livermore, CA 94550

Correspondence:

Robert J. Meagher : rmeaghe@sandia.gov

Aashish Priye: apriye@sandia.gov

Supporting information contains:

1. **Supplementary figure: Fig. S1**
2. **Supplementary figure: Fig. S2**
3. **Supplementary figure: Fig. S3**
4. **Supplementary figure: Fig. S4**
5. **Supplementary figure: Fig. S5**
6. **Supplementary table: Table S1. List of primers designed in this study**
7. **Supplementary note: Primer design**
8. **Supplementary note: Dried reagent formulation**

Supplementary Figures

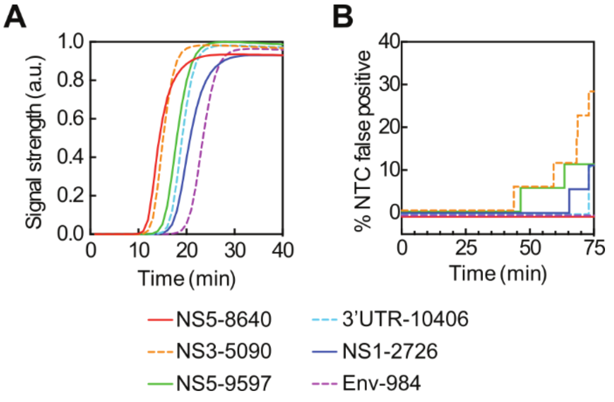

Fig. S1. The NS5-8640 primer set is both the fastest and least error-prone RT-LAMP primer set we tested for Zika. These initial, unoptimized RT-LAMP reactions were conducted with a standard 8 mM $MgSO_4$ at 65°C. A) Averaged (n = 3) real-time RT-LAMP detection of 10^3 PFU equivalent/mL extracted ZIKV RNA by SYTO 9 for each primer set. Real time signal is normalized to the maximum achieved signal for any primer set. B) An extended incubation of 75 minutes was run to encourage false positive generation by spurious amplification in NTC reactions (n = 18 per primer set). The % of reactions generating false positives, detected as total dsDNA synthesis by SYTO 9 (not QUASR), are shown over time. Curves have a slight vertical offset to aid in visualization.

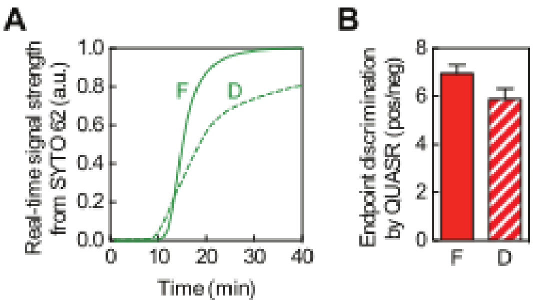

Fig. S2. QUASR RT-LAMP assays are readily stabilized in a dry format using reagents from Biomatrica, Inc. A) Averaged (n = 7) real-time RT-LAMP detection of 10^2 PFU/mL intact ZIKV by SYTO 9. F = Fresh reagent mixture with betaine; D = dried reagents with preservation mixture without betaine. B) Endpoint discrimination in dried RT-LAMP assays by QUASR is clear-cut and compatible with dried preservation. Error bars indicate standard deviation (n = 7).

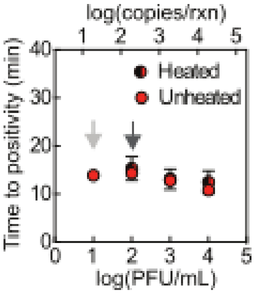

Fig. S3. Heating intact ZIKV prior to real-time detection with RT-LAMP reduces assay sensitivity. Time to positivity is shown versus concentration of intact ZIKV. Error bars indicate standard deviation (n = 4). While detection speed doesn't change appreciably, only 3/4 heated reactions turned positive at 10^2 PFU/mL (dark gray arrow). 1/4 unheated reactions and 0/4 heated reactions turned positive at 10 PFU/mL (light gray arrow).

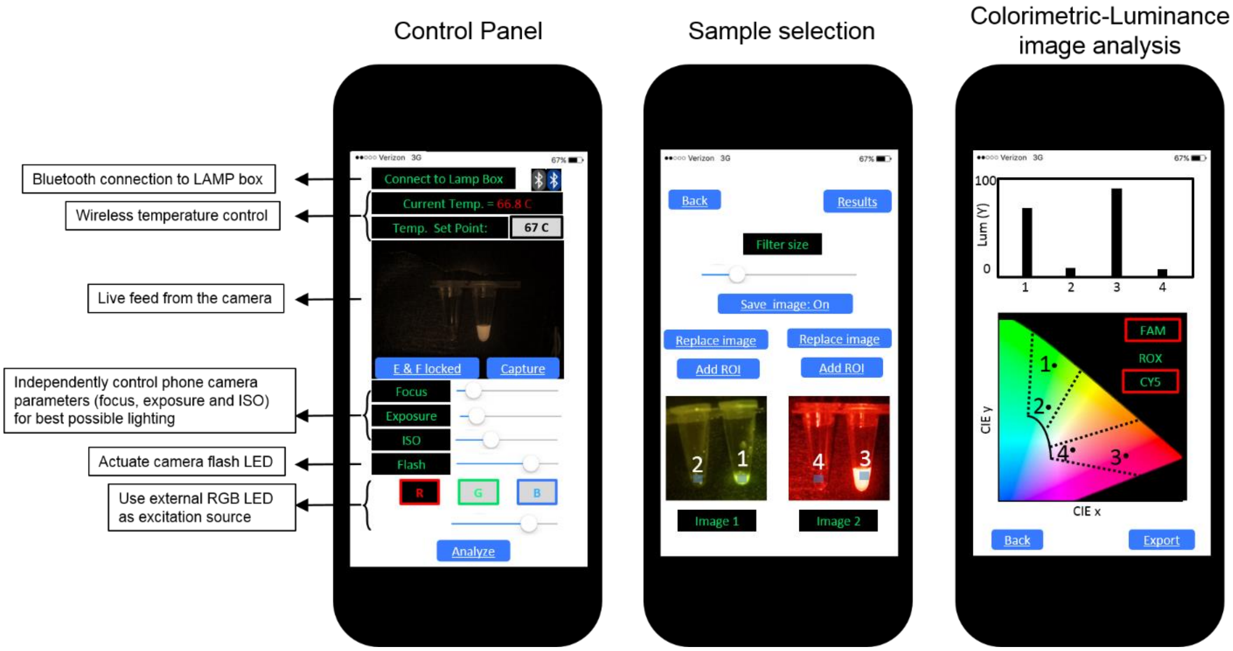

Fig. S4. LAMP2Go application screenshots. The app starts with the "control panel" screen where the user establishes a connection with the LAMP box, monitors and controls the heater temperature, tunes the camera parameters and actuates the RGB LED source to illuminate the assay. Before and after reaction images taken on this screen are then passed on to the "sample selection" screen where the user can conveniently choose as many regions of interest (ROI) within the acquired images. Finally, the acquired ROIs are analyzed and the results are displayed on the "Colorimetric –Luminance image analysis screen".

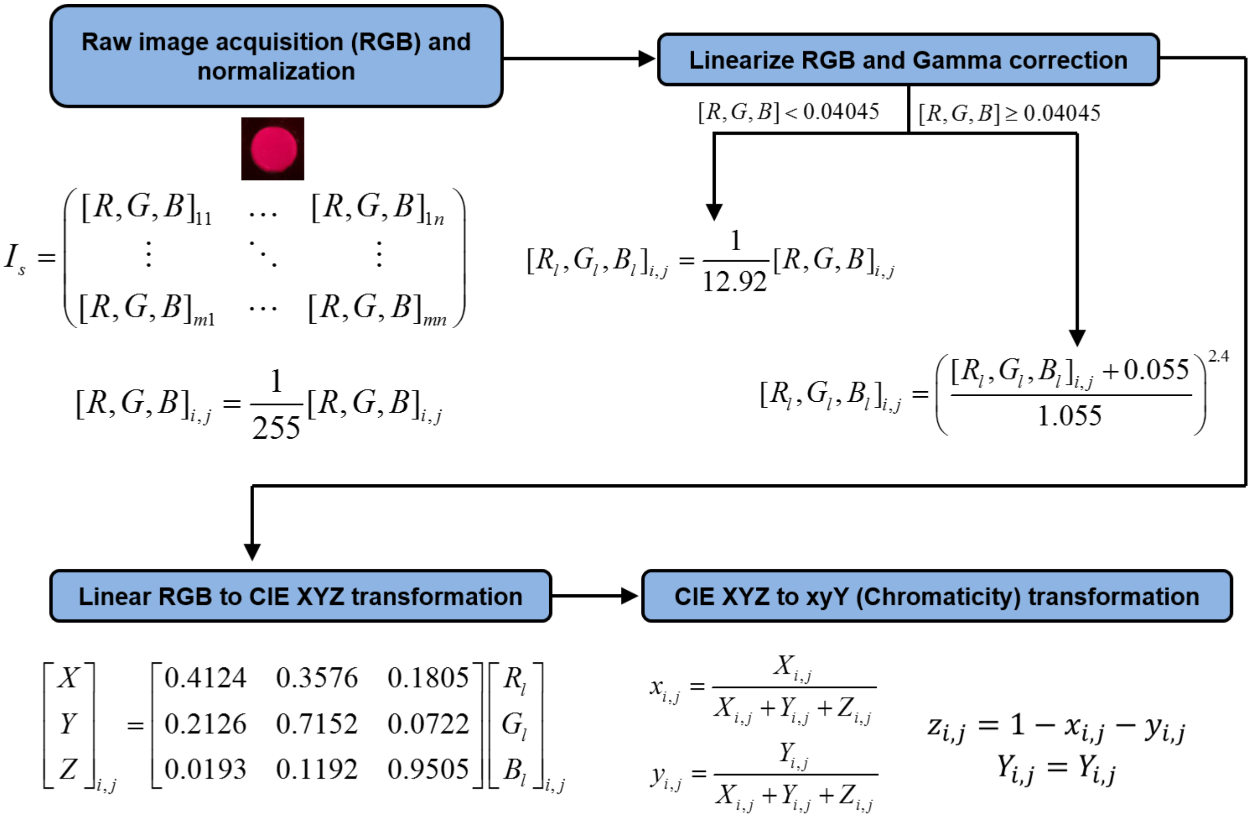

Fig. S5 Colorimetric analysis flow chart. The RGB image acquired from the CMOS sensor is first normalized to value between values 0 and 1 after which they are linearized with gamma correction based on the intensity of individual pixel colors. The linearized RGB matrix is then transformed into the CIE XYZ values which can then be normalized to represent the color and luminance value in terms of two chromaticity coordinates (x and y) and Y respectively. i and j denote the pixel coordinates.

Table S1. List of primers designed in this study

Target region	Primer	Sequence (5' → 3')
Env-984	F3	GCATAGGAGTCAGCAATAGG
	B3	TCTGAYTGCTTGTCAAGGTAG
	LF	AACCTCCATGTTCCAAGACAA
	LB	AGATCCTACTGCTATGAGGCA
	FIP	GTCCTGTGCCATYACGGTAACATGTGGAAGGTATGTCAGGT
	BIP	AGTCAGCAACATGGCGGAGGAAGCCATGTCYGATATTGA
NS1-2726	F3	GAAGAGAATGGAGTTCAACTGA
	B3	ATGATCCTCCACAAGAAAGC
	LF	GGCAATCTCTGTGGACCTC
	LB	GTCGTGGATGGTGACACA
	FIP	AGCTCGTTCACAGGCACGGTCGTTGTGGGATCTGTAAA
	BIP	TTCGTCAGAGCAGCAAAGACAATGTTTGAGTGGGCATTCC
NS3-5090	F3	GCATAGGAGTCAGCAATAGG
	B3	TCTGAYTGCTTGTCAAGGTAG
	LF	AACCTCCATGTTCCAAGACAA
	LB	AGATCCTACTGCTATGAGGCA
	FIP	GTCCTGTGCCATYACGGTAACATGTGGAAGGTATGTCAGGT
	BIP	AGTCAGCAACATGGCGGAGGAAGCCATGTCYGATATTGA
NS5-8640	F3	TTGTCAGGCTCCTGTCAA
	B3	TTGCTACGAACCTTGTTGAT
	LF	TGCTGACCATACGGTGTG
	LB	GGTCTCTTCCTGGTTGTGG
	FIP	TGGCACCCTAGTGTCCACTTTTTAGGAATAGCCATGACCGA
	BIP	CAAGAAGGCACTCGTCAGGTTTTGTGTTTGCCTAGCTCTTT
NS5-9597	F3	AAGTGACCAACTGGTTGC
	B3	ATCAGTTCATCTTGGTGGC
	LF	CAATTGGCTTCACAACGCA
	LB	GGGACAACTGGGAAGAAGTT
	FIP	TTCAAGAACCTGAGGGCATGTGTTTTATGGCAGTCAGTGGAGAT
	BIP	CACACAAGAGTGGAAACCCTCAATGGAGCTTGTTGAAGTGG
3'UTR-10406	F3	CTAGTCAGCCACAGCTTG
	B3	CTAACCACTAGTCCCTCTTCT
	LF	AGCATGGCTTCTTCCGTG
	LB	CCTTCCCCACCCTTCAATC
	FIP	TGACTCAGTGTCCTCTGAGGGTTTTCCTATAGTCAGGCCGAGAA
	BIP	GAGGCGCAGGATGGGAAACACAGCTGATCTCCAGTTC

Supplementary note: Primer design

The selected primers were subjected to the following (manual) tests:

(1) Comparison against the sequence alignment: Ideally all primers would be a perfect match to all sequences in the alignment, although for some sets we allowed for a single mismatch in a minority of sequences in the alignment.

(2) Checking the complete set of primers (including full-length FIP and BIP) for self-dimerization or cross-dimerization using the Multiple Primer Analyzer tool from ThermoFisher Scientific (https://www.thermofisher.com/us/en/home/brands/thermo-scientific/molecular-biology/molecular-biology-learning-center/molecular-biology-resource-library/thermo-scientific-web-tools/multiple-primer-analyzer.html) using highest sensitivity settings. Generally, we reject any primer set that has an amplifiable primer dimer (with more than 3-4 base complementarity at the 3' end of a primer), or other long regions of complementarity between primers.

(3) Checking each primer (including full-length FIP and BIP) for hairpin structures using mFold tool from Integrated DNA Technologies (www.idtdna.com), with default settings except adjusting Mg++ concentration to 8 mM (the default LAMP condition, prior to optimization). Generally, we reject any primer set if the primer forms a self-amplifying hairpin structure (with complementarity at the 3' end), or excessively high melting hairpins (although FIP and BIP primers with hairpins stable up to 65 °C can work, as long as they are not amplifiable). During this step we also screen the loop primers and inner primers for suitability for QUASR. This screening consists of selecting primers not having a hairpin occupying the 5' end of the primer, with T_m greater than about 45 °C. At this stage we can consider adding a short linker (e.g. between 1 and 4 "T" bases) between the two regions of the inner primers (e.g. between F1c and F2) as this is sometimes successful in disrupting hairpins, and such linkers are a common feature of LAMP primers that is neither necessary or deleterious.

(4) Checking (via BLAST) the complementarity of the primer set to all Zika virus sequences in the database (obviously matching most Zika is desirable), as well as checking (via BLAST) the complementarity of the primer set to all "NOT Zika virus" sequences in the database to identify any likely cross reactivity. A primer set would be rejected at this stage if more than one of the primers was a close match for the same non-target virus, although allowance would be made for highly unrelated organisms (e.g. partial homology to a marine bacteriophage that is unlikely to be found in a human clinical sample would be acceptable, whereas close homology to West Nile virus would be grounds for rejection).

Primer sets meeting these criteria were synthesized at small scale (without QUASR modification) and screened prior to scaling up synthesis for the best performing sets.

Supplementary note: Dried reagent formulation

The dried reagent formulation consisted of a proprietary stabilization mixture from Biomatrica Inc, 0.14 mM of each nucleotide, 2 µM SYTO® 9 Stain (Thermo Fisher Scientific), 2 units of Porcine RNase Inhibitor[1], 0.2 µM each F3 and B3, 0.8 µM each LF and LB, 1.6 µM each FIP and BIP primers (Table S1), 3.2 units of avian myeloblastosis virus (AMV) reverse transcriptase (Life Sciences Advanced Technologies Inc., St Petersburg, FL), and 3.2 units of Bst 2.0 WarmStart® DNA Polymerase (NEB). The reaction mixture was dried down and stored. At the time of reaction, a rehydration mixture containing 1x Isothermal Amplification Buffer (20mM Tris-HCl, 10mM $(NH_4)2SO_4$, 50mM KCl, 2mM $MgSO_4$, 0.1% Tween® 20, pH 8.8@25°C, NEB) and 8 mM $MgSO_4$ were added along with water. Betaine was not added while rehydrating the dried reagents.

References:

1. Guo, W., et al., *High level soluble production of functional ribonuclease inhibitor in Escherichia coli by fusing it to soluble partners.* Protein expression and purification, 2011. **77**(2): p. 185-192.
